# More Than a Buffer in Biochemistry: Tris as an Architect and Gatekeeper of Metal–Oxo Assembly

**DOI:** 10.1002/anie.202525355

**Published:** 2026-02-24

**Authors:** Nadiia I. Gumerova, Annette Rompel

**Affiliations:** ^1^ Universität Wien Fakultät für Chemie Institut für Biophysikalische Chemie Wien Austria

**Keywords:** Keggin polyoxometalate, metal‐oxides, THAM, Tris buffer, tromethamine

## Abstract

Polyoxometalates (POMs, molecular metal–oxo clusters) are typically studied and applied in aqueous media, where routine buffers determine which clusters form, persist, or react. Tris(hydroxymethyl)aminomethane (Tris) is a ubiquitous buffer near physiological pH and has produced outcomes that pH control alone could not explain, prompting a simple question with large consequences: What does Tris actually do to POMs? Quantification across several POM families shows that this loose buffering correlates with longer lifetimes of intact anions, the formation of Tris‐specific adducts, and tunable stability through ionic‐strength adjustment. We describe three molecular roles of Tris. First, Tris acts as an alkoxy donor that embeds μ‐O─CH_2_ units within POM scaffolds. Second, Tris functions as a chelator that arrests early tungsten‐oxo condensation and stabilizes a minimal isopolytungstate. Third, Tris serves as a structure‐directing medium, since a chromium‐incorporated Keggin forms only in Tris buffer at pH 7.5 and displays single‐ion‐magnet behavior. We advocate a speciation‐first workflow that logs attained pH, reports buffer identity, concentration, and ionic strength, and verifies species by orthogonal spectroscopic, diffraction, and computational methods. The implication extends well beyond POM chemistry: in catalysis, electrochemistry, and biomaterials, buffers and amine additives can redirect speciation, alter redox access, and bias kinetics.

## Graphical abstract

Tris(hydroxymethyl)aminomethane, depicted as an octopus, acts as an alkoxy ligand, chelator, and structure‐directing buffer that programs polyoxometalate's speciation, nucleation, and heterometal insertion in aqueous solution.

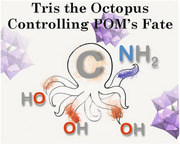

## 1 Introduction

Buffers are among the most frequently “invisible” reagents in chemical biology, yet they often decide the fate of reactions in water [1, 2] Tris (tris(hydroxymethyl)aminomethane, tromethamine) is emblematic: it is inexpensive, ubiquitous, and, crucially, has a p*K*_a_ of ∼8.1‐8.3 near room temperature, making it ideal for maintaining near‐neutral to mildly basic pH (≈7–9) where many biological processes operate. The prominence of Tris extends far beyond benchtop biochemistry. Modern mRNA vaccine formulations explicitly employ tromethamine/tromethamine·HCl (Tris/Tris‐HCl) as buffering components (e.g., Spikevax, Moderna) or Tris‐sucrose compositions (e.g., Comirnaty “gray‐cap” presentations), underscoring its role in stabilizing sensitive biologics at cold‐chain compatible storage and transport temperatures [3]. Despite its popularity, Tris is not a Good's buffer [4] and has several problems: it likely helped accelerate Good's buffer development because its primary amine uncouples electron transport from ATP synthesis [1] and its temperature coefficient is large (≈ –0.03 pH units °C^−^^1^). Tris can increase *Escherichia coli* outer‐membrane permeability [5] and interact strongly with the protein backbone (sometimes stabilizing proteins, implying ligand‐like behavior) [6].

**FIGURE 1:**
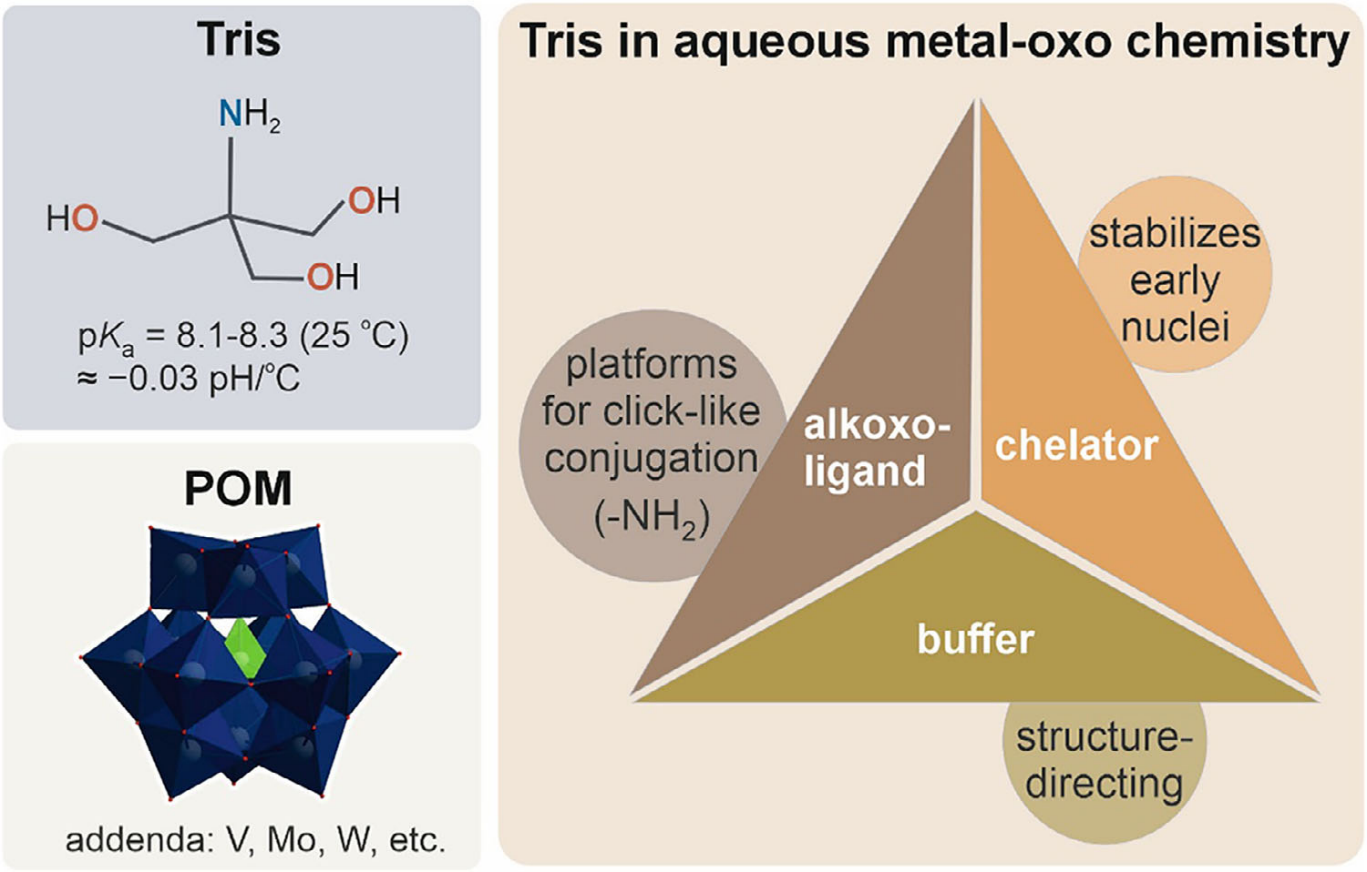
Tris in metal–oxo chemistry: structure, roles, and reach.

Yet Tris is not chemically innocent. Its primary amine and triol motif can coordinate metal ions (Figure 1). In systems containing transition metals, Tris can act as a competent ligand/chelator, altering speciation and even catalysis. Contemporary kinetic and thermodynamic studies show that Tris binds Cu^2+^ measurably, forming defined complexes whose presence depends on concentration and pH, and can thereby influence rates in Cu‐peptide and metalloenzyme assays [7]. Mechanistically, this is consistent with basic coordination‐chemistry principles: N/O‐donor ligands donate electron density into accessible *d* orbitals of transition‐metal cations to form coordination complexes. The practical lesson is simple but often overlooked: buffer components can be ligands, and even low levels of buffer–metal complexes may bias outcomes in “metal‐dependent” experiments.

Running in parallel, polyoxometalates (POMs), molecular metal–oxo coordination compounds of Mo, W, V, Nb, and so on, have become versatile modules from catalysis to medicine (antibacterial [8, 9, 10, 11], antiviral [12, 13], and anticancer [14] substances). A defining feature of POMs in water is dynamic speciation: the identity and distribution of anions depend on pH, concentration, ionic strength, counterions, and additives. Over the past few years, systematic maps — the Speciation Review (2020) [15] and the Speciation Atlas (Part I 2023 [16], Part II 2025 [17]) — have established that the POM you think you made may not be the POM present under application conditions. For any POM aimed at biological media, where buffers and amines abound, speciation‐aware design becomes imperative.

Tris itself participates in POM chemistry. Historically, Tris(hydroxymethyl)aminomethane was already used as a base to generate the hexalacunary Wells–Dawson anion [*α*‐H_2_P_2_W_12_O_48_]^12−^ from [*α*‐P_2_W_18_O_62_]^6−^, representing one of the earliest explicit applications of Tris in POM synthesis, although its potential non‐innocent role was not yet discussed [18]. Beyond pH control, Tris can arrest nucleation and stabilize unusually small isopolyoxo species (i.e., isopolyoxometalate anions composed solely of addenda metals and oxo ligands, without a heteroatom template) [19], serves as a tripodal triol that caps faces of certain archetypes (e.g., Anderson–Evans, Lindqvist, Wells–Dawson) [20, 21, 22, 23] or direct specific synthesis routs [24]. In other words, Tris toggles among buffer, ligand, and counterion/solvation modulator—three roles that can cooperate or conflict depending on the applied conditions. For POMs in medicine, these Tris’ roles matter. When POMs are evaluated for activity (e.g., protein binding, cellular uptake, antimicrobial/anticancer assays), the medium composition with Tris, can reshape cluster identity, charge state, and surface chemistry, thereby altering measured bio‐effects [16]. The community now recognizes that rigorous speciation control (pH logging, ionic‐strength management, orthogonal analytics such as multinuclear NMR/ESI‐MS/in‐situ UV–vis) is a prerequisite for reproducible claims and for comparing results across labs.

Here it is argued that Tris is a design element, not an incidental additive, and analyzed along three actionable axes: (i) triol functionalization (how Tris furnishes click‐ready hybrid POMs with modular organic handles); (ii) nucleation/stabilization (how Tris arrests early condensation to yield otherwise inaccessible POMs); and (iii) interfacial/counterion effects (how Tris/TrisH^+^ tune solvation, assembly, and biointerfaces). This perspective is coupled with best‐practice guidance for speciation‐first workflows and discusses opportunities in medicine where Tris–POM chemistry could be leveraged or must be controlled to ensure that structure, function, and medium are aligned. Although framed around POMs, these rules generalize to other metal‐oxo clusters, metal‐organic frameworks (MOFs)/sol–gel networks, and nanoparticle syntheses, where amine buffers steer speciation, redox windows, and assembly [25, 26].

## 2 Embedding μ‐O–CH_2_ Units: Tris Alkoxylation in POM Cores

Tris and related triols have enabled face‐selective grafting across several archetypes, with Anderson–Evans clusters [27] emerging as a particularly versatile platform. The first triol‐grafted Anderson–Evans polyoxomolybdate (POMo) was synthesized by Hasenknopf 2002 [28], inaugurating a family of Anderson–Evans cores single‐ or double‐side hybridized with triol ligand [29]. Beyond Anderson–Evans, tris‐alkoxo derivatization has been demonstrated on Lindqvist [30], Wells–Dawson [31], and Keggin [32] families [33]. Functionalization of non‐Anderson‐Evans frameworks with tripods was largely confined to mixed addenda systems, where anchoring often occurs at {V_3_O_13_} [33] fragments (Wells‐Dawson/Keggin), embedded metal cores (e.g., {Ni_6_PW_9_} [34]) or installing M─O─X linkages (M = Mo, W; X = Si, P, As, Sn, N) at terminal oxygens [33]. Collectively, alkoxylation has become a go‐to strategy because triols offer diversity and tunability, and the resulting hybrids underpin applications from bio‐hybrids and supramolecular assembly to charge storage, MOFs, nanostructures, and photochemistry. Triol‐grafted POMs, including those bearing Tris‐derived ligands, also provide post‐functionalizable organic termini that can be elaborated further by amide/carbamate coupling, metal–organic framework formation, or supramolecular host–guest strategies, which have been comprehensively reviewed elsewhere [33], and we refer the reader to that work for a broader overview of hybrid POM post‐functionalization.

**FIGURE 2:**
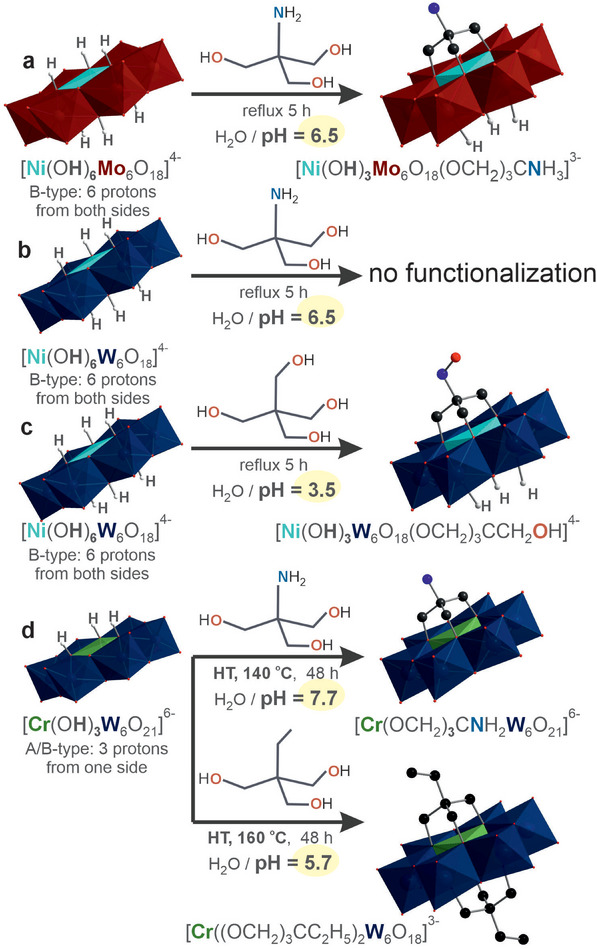
Unlocking Tris alkoxylation on Anderson‐Evans POTs. (a) Successful Tris attachment on {NiMo_6_} core; (b) {NiW_6_} resists Tris attachment: no W─O─C formation observed in water at pH 6.5; (c) alkoxo‐functionalization of {NiW_6_} under acidic conditions with pentaerythritol; (d) direct single‐ and double‐side alkoxo capping on [Cr(OH)_3_W_6_O_21_]^6−^ under hydrothermal (HT) conditions. Color code: {MoO_6_}, red octahedra; {WO_6_}, dark blue octahedra; {NiO_6_}, teal octahedra; {CrO_6_}, green octahedra; O, red; N, blue, C, black; H, white.

Despite the broad success of tris‐alkoxo grafting on molybdate and vanadate systems, extending this logic to polyoxotungstates (POTs) has been difficult because μ‐O bridges in {WO_6_} are comparatively inert. This inertness is largely kinetic and electronic in origin: in *d* ^0^ W(VI), W─O bonds are shorter and more covalent than in the Mo(VI) and V(V) analogues, the bridging oxygens are less basic, and ligand exchange at tungsten is slower. As a result, protonation and nucleophilic substitution at μ‐O are disfavored and require more forcing conditions. The first systematic test asked whether a tripodal triol–amine could directly graft onto a B‐type (fully protonated central {XO_6_}) tungsten Anderson‐Evans anion: [Ni(OH)_6_W_6_O_18_]^4−^ [35]. Under the acidic conditions required for {NiW_6_} alkoxylation (reflux, pH ≈ 3.5), Tris was intrinsically incompatible: its basic amine neutralized the medium, and excess ligand could not be used without raising pH beyond the window needed for W–μ‐O activation. Consequently, no Tris grafting was achieved on [Ni(OH)_6_W_6_O_18_]^4−^. Switching to pentaerythritol, a triol lacking –NH_2_, restored the required acidity (pH ≈ 3.5) and enabled triol functionalization of the tungsten Anderson core – [Ni(OH)_3_W_6_O_18_(OCH_2_)_3_CCH_2_OH]^4−^ [35] (Figure 2). In sharp contrast, Tris readily attaches to the molybdate analogue {NiMo_6_} in water at ∼pH 6.5 [36], a regime too basic for tungsten, underscoring the element‐dependent pH window for μ‐O alkoxylation.

Then the heterometal and face‐hydroxo pattern was shifted. In 2019 [20], it was shown that the mixed‐type Anderson POT [Cr(OH)_3_W_6_O_21_]^6−^, an intermediate between A‐ and B‐types with a Cr^3+^ center bearing three μ_3_‐OH and three μ_3_‐O sites (Figure 2), does undergo direct triol functionalization with Tris in water under more forcing hydrothermal conditions relative to reflux, yielding single‐ and double‐face Tris‐capped hybrids. Based on this result, several mechanistic conclusions follow:

- Pre‐protonation is not a prerequisite. Contrary to the “B‐type‐only” ({NiW_6_} or {NiMo_6_} in Figures 2a–c) dogma, the Cr–W_6_ platform permits access to the A‐side under mild aqueous conditions (Figure 2d). This is attributed to the μ_3_‐OH presence and enhanced polarization of the μ‐O network around Cr^3+^, lowering the barrier to μ‐O attack on Tris (dehydrative condensation) without externally pre‐charging the face.
- Second‐face capping is facilitated once the first Tris is installed. The bis‐capped product forms under conditions close to the mono‐capped synthesis, but at elevated temperature.
- Reactive role of Tris versus “innocent” buffer. Tris acts as a true alkoxy donor (triol) rather than merely a base or counterion. The amine remains available as a post‐functionalizable handle (–NH_2_), enabling amide/carbamate chemistry on the hybrid POM without compromising the core.

## 3 Tris as a Chelator: Arresting Early Tungsten Condensation and Retaining the Minimal Iso‐W Core

**FIGURE 3:**
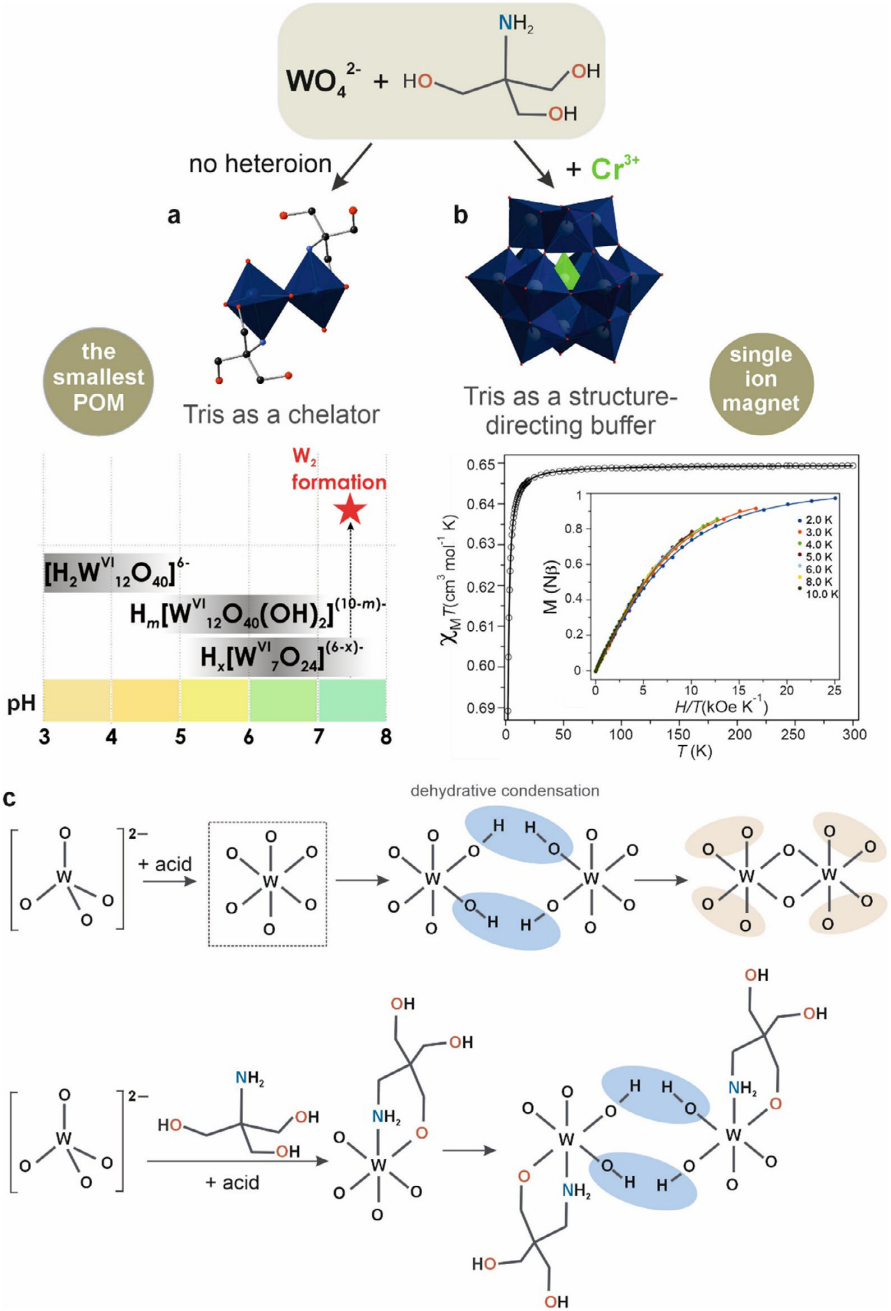
Tris as a chelator and director in POT self‐assembly. (a) Tris as a chelator: coordination and alkoxylation of early tungsten‐oxo fragments yield the minimal iso‐W unit (W_2_) with μ‐O─CH_2_ linkages within the scaffold. Bottom left: pH corridor summarizing POM speciation, highlighting the near‐neutral window where W_2_ is present. *x* in H_*x*_[W^VI^_7_O_24_]^(6−*x*)−^ is 0, 1; *m* in H_*m*_[W^VI^_12_O_40_(OH)_2_]^(10−*m*)−^ between 0 and 3. (b) Tris as a structure‐directing buffer: in the presence of Cr^3+^, Tris selects a Cr‐incorporated Keggin accessible only in Tris solution. Bottom right: χ_M_T versus T confirms single‐ion‐magnet behavior of the embedded tetrahedral Cr^3+^ center [24]. (c) Schematic comparison of early tungsten‐oxo condensation with and without Tris. Up: classical growth from monomeric [WO_4_]^2−^ toward larger isopolytungstates; bottom, coordination and alkoxylation of early tungsten‐oxo fragments by Tris yield the minimal iso‐W unit ({W_2_}) with μ‐O─CH_2_ linkages within the scaffold, arresting further condensation. In the beige circles, there are additional opportunities for further condensation. The nitrogen donor as depicted as neutral NH_2_ consistent with the coordination through the lone pair to W. Color code: {WO_6_}, dark blue octahedra; {CrO_6_}, green octahedra; O, red; N, blue.

Tris couples Brønsted buffering with hard O/N coordination. In a 2021 study [19], the smallest polyoxotungstate retained by Tris stabilization [W_2_O_6_(C_4_O_3_NH_10_)_2_]^2−^ was isolated, demonstrating that Tris does not merely set pH value it coordinates and caps nascent tungsten‐oxo fragments, arresting further condensation and preserving a minimal iso‐W scaffold in water (Figure 3a). What does “chelation” mean here? At near‐neutral pH, deprotonation of one or more Tris hydroxyls enables alkoxy binding to W(VI) (μ‐O─CH_2_─ linkages). At the same time, the amine can engage via hydrogen bonding/outer‐sphere ion pairing or, depending on local microacidity, form inner‐sphere contacts that help close a chelate loop around a small W‐oxo nucleus (Figure 3a). The result is a kinetically persistent, coordinatively saturated micro‐cluster that resists the usual growth to Lindqvist/Keggin sizes.

Key experimental signatures from the 2021 work include structure‐level evidence: crystallography locates Tris‐derived alkoxy oxygens bound to tungsten within the cluster, with connectivity incompatible with mere counterion effects. IR spectra show emergent C─O (alkoxy) bands alongside the tungsten–oxo fingerprint, consistent with genuine grafting rather than hydrogen bonding. In solution, ^183^W NMR, Raman spectroscopy, ESI‐MS, and DFT together indicate that the {W_2_} core is unstable at pH 7.5 in water; nevertheless, ESI‐MS reveals intermediates featuring alternative Tris attachment modes that are at least partially present under slightly acidic conditions. Figure 3c summarizes a qualitative mechanistic picture of how chelation competes with growth. In slightly alkaline tungstate solutions without Tris, monomeric [WO_4_]^2−^ units condense via μ‐O‐bridged dimers and small oligomers toward larger isopolytungstate frameworks such as Lindqvist‐ and Keggin‐type species through a sequence of olation/oxolation steps. Along this pathway, the dimeric {W_2_} motif is transient: terminal W═O groups and μ‐O sites remain available for further W─O─W bond formation, so condensation continues downhill in free energy. In the presence of Tris, deprotonated –CH_2_O^−^ groups attack electrophilic W centers at this early stage, replacing would‐be W─O─W bridges with W─O─CH_2_– linkages and closing chelate loops around a {W_2_} core. This multidentate wrapping both saturates coordination and redistributes charge, raising the barrier for additional condensation and effectively “freezing” the condensation ladder at the {W_2_} level. Although we do not claim a fully resolved step‐by‐step mechanism, this interception picture is consistent with the observed instability of {W_2_} in Tris‐free solution.

Why this matters extends well beyond isolating a curiosity: (i) synthetically, Tris provides a handle to trap and interrogate early intermediates along the tungsten‐condensation pathway, sharpening mechanistic understanding and enabling designed access to low‐nuclearity species; (ii) in formulations and biological media, where Tris is often present, chelation‐stabilized micro‐clusters may persist and bias observed reactivity or biological responses—an object lesson that the buffer is a reagent; (iii) and as a platform, the embedded –NH_2_ of Tris survives grafting, furnishing a post‐functionalizable handle (amide/carbamate chemistry) on a uniquely small, well‐defined inorganic core. Practically, tight pH control, sufficient excess Tris to secure chelation, and verification of outcomes with orthogonal analytics are essential, while avoiding phosphate or other competitive ligands that can out‐chelate or redirect speciation.

## 4 Tris as a Structure‐Directing Buffer

The previous section showed that Tris can chelate and arrest early condensation of tungsten oxo fragments. When a heterometal is present, however, the trajectory of POM assembly can change altogether: Tris may behave innocuously as a counter cation, stabilizing a preformed framework without altering its topology (e.g., TrisH^+^ salts of known POMs) [37], or, in distinct cases, it can direct nucleation and speciation toward otherwise inaccessible architectures. The first, outer‐sphere scenario is exemplified by crystal structures in which Tris appears only as protonated TrisH^+^ balancing charge and building hydrogen‐bond networks around classical POM anions [38]. It is also illustrated by decavanadate salts of the type (TrisH)_4_[H_2_V_10_O_28_]·10H_2_O and (TrisH)_6_[V_10_O_28_], in which TrisH^+^ enhances oxide solubility and templates the packing of [V_10_O_28_]^n−^ without forming any M─O─C linkages [39]. In Wells–Dawson systems, outer‐sphere TrisH^+^ can even promote linear poly‐POM chain formation through hydrogen bonding and electrostatics rather than covalent functionalization [40]. In contrast, our Cr–Keggin case demonstrates the second, structure‐directing regime [24].

In 2020 [24], we reported that incorporation of Cr^3+^ into a Keggin polyoxometalate stabilizes a labile {CrO_4_} tetrahedral unit inside the anion and gives rise to single‐ion magnet behavior. Crucially, the targeted Cr–Keggin formed only in Tris buffer at pH 7.5 (Figure 3b). Repeated attempts in alternative media did not yield the same architecture. This exclusivity points to Tris acting as a structure‐directing buffer rather than a passive participant. We hypothesize that Tris simultaneously (i) sets a precise pH window that balances hydrolysis/condensation of both W and Cr aquo species, (ii) transiently ligates or solvates Cr^3+^ (outer‐sphere H‐bonding/ion‐pairing or weak inner‐sphere contacts), smoothing the assembly of a tetrahedral {CrO_4_} fragment within the Keggin cavity, and (iii) modulates ionic strength and hydrogen‐bond networks to favor packing and growth of the specific heteropolyanion over competing W‐oxo condensates. Under these conditions, the heterometal identity, its hydrolysis kinetics, and the Tris‐defined medium conspire to select a single structural solution, Cr‐incorporated Keggin, that we could not realize outside Tris. The outcome underlines a general design principle: buffers with donor/acceptor functionality and well‐placed p*K*_a_ values can gate which heteroatom‐doped POM architectures nucleate and persist, even when they leave no covalent trace in the final structure.

Beyond synthetic novelty, the Cr‐centered Keggin exhibits genuine single‐ion magnet (SIM) behavior, confirming that it is not a mere crystallographic curiosity (Figure 3b). The tetrahedral Cr^3+^ (S = 3/2) embedded in the rigid Keggin oxygen cage experiences a low‐symmetry ligand field that generates appreciable zero‐field splitting and magnetic anisotropy, enabling slow magnetic relaxation observable in ac susceptibility (out‐of‐phase χ″ signals) under appropriate conditions. Due to the fact that the Cr center is magnetically isolated (negligible exchange pathways through the oxide framework), the relaxation dynamics primarily reflect single‐ion physics (Orbach/Raman pathways modulated by the local distortion), providing a clean structure–property link: the Tris‐directed assembly that stabilizes the {CrO_4_} unit is precisely what endows the molecule with SIM characteristics. This magnetism elevates the complex from a formulation‐dependent oddity to a functional molecular material with relevance to information storage and spin‐based devices.

## 5 Tris as a Buffer

**FIGURE 4:**
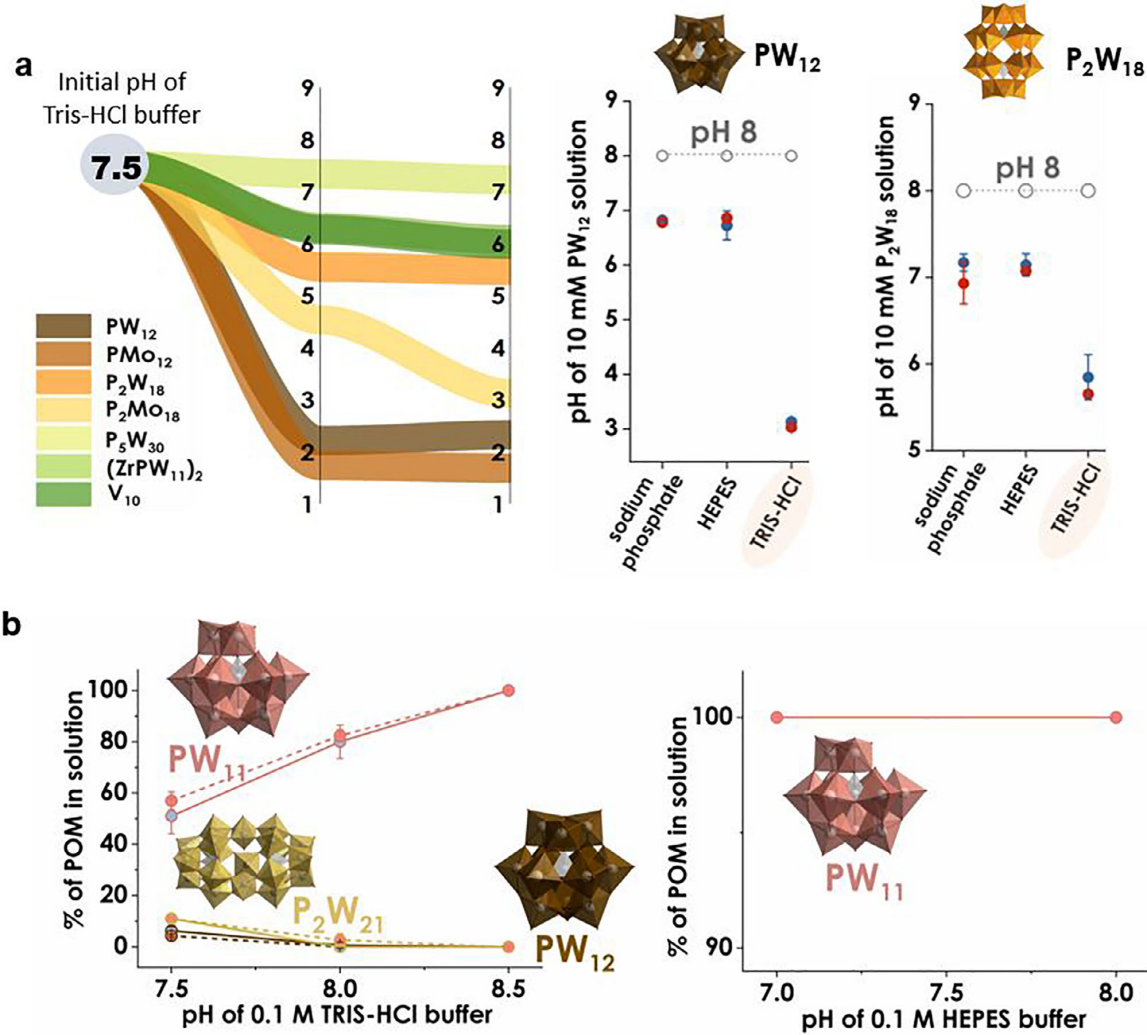
Tris‐driven pH drift and Tris‐specific speciation. (a) Left: Starting from 0.1 M Tris–HCl, pH 7.5, the attained pH after dissolving 10 mM POM depends strongly on the anion, with drops up to ∼5 pH units, indicating buffer engagement during hydrolysis/condensation. Right: Comparison at nominal pH 8 shows larger pH shifts in Tris–HCl than in HEPES or sodium phosphate for [*α*‐PW_12_O_40_]^3−^ (PW_12_) [32] and [*α*‐P_2_W_18_O_62_]^6–^ (P_2_W_18_) [31]. (b) In Tris–HCl, the fraction of parent POM in solution varies with the *attained* pH and reveals Tris‐specific products/adducts (e.g., [PW_11_O_39_]^7−^ (PW_11_) and [P_2_W_21_O_71_(H_2_O)_3_]^6−^ (P_2_W_21_)) [41] that increase with pH, whereas in HEPES the speciation appears largely unchanged. Color code: {WO_6_}, dark blue octahedra; {CrO_6_}, green octahedra; O, red; N, blue. Part b of the figure is adopted from [16].

Having shown that Tris can chelate and even direct assembly, Tris is now treated purely as a buffer and it is investigated how it reshapes speciation under application conditions. In the context of POM chemistry, “speciation” refers to the full distribution of polyoxoanions present in solution, rather than a single idealized composition. A nominal “PW_12_” sample, for example, can contain intact Keggin ions, partially lacunary derivatives, smaller fragments, and buffer‐ or counterion‐stabilized adducts, all interconverting via protonation/deprotonation and condensation/fragmentation reactions [41]. Which species dominate is controlled by pH, concentration, ionic strength, counterions, and additives such as buffers. Speciation maps or atlases, therefore, track how the relative populations of parent, lacunary, and fragment species respond to changes in key speciation parameters, providing a direct link between the nominal experimental conditions and the anions that are actually present under application conditions [15, 16, 17]. In our speciation atlas [16], ten archetypal POMs were surveyed across eight common buffers (pH 3–9; uniform temperature/ionic strength) and their solution behavior was tracked. A conspicuous outlier was 0.1 M Tris–HCl: at “reasonable” POM loadings (≈10 mM), Tris failed to hold pH constant, and critically, the pH drift was POM‐dependent (Figure 4). For hydrolysis‐prone families (e.g., Keggin and certain Wells–Dawson members), dissolution in Tris could drop pH by up to ∼5 units, whereas more robust frameworks produced smaller excursions (Figure 4). This behavior is not a trivial experimental nuisance: it is a speciation driver that moves systems through distinct condensation/fragmentation windows during the first minutes of contact.

Paradoxically, greater persistence of intact Wells–Dawson anions was observed in Tris than in “stronger” buffers such as phosphate or HEPES (4‐(2‐hydroxyethyl)‐1‐piperazineethanesulfonic acid) at nominal pH 8 (Figure 4). The explanation is engagement over isolation. Tris buffers less rigorously, and it interacts by proton transfer, H‐bonding, outer‐sphere association, and, in some cases, inner‐sphere alkoxylation, thereby dampening uncontrolled hydrolysis and slowing conversion to lacunary or degraded forms. In other words, buffer capacity alone is a poor predictor of POM integrity: a chemically “softer,” interactive medium can stabilize parent anions longer than a tightly clamped pH that offers no compensating interactions. Those interactions are observable. In Tris, several POM families form unique adducts or mixed species that are absent in HEPES or sodium phosphate buffers under otherwise identical conditions. This is the practical meaning of “the buffer is a reagent”: the observed product distribution reports on POM–buffer chemistry, not just on nominal acidity.

From an acid–base perspective, Tris and HEPES are often treated as interchangeable near‐neutral buffers. Our results show that they are functionally similar as buffers but chemically and biologically different media: in the systems studied here, HEPES behaves largely as an outer‐sphere spectator, whereas Tris can act as an N/O‐donor ligand, chelator, and, in some cases, alkoxy source. Piperazine‐based hydroxyalkyl buffers such as N,N‐bis(2‐hydroxyethyl)piperazine, which combine a rigid chelating topology with hydroxyethyl substituents, are therefore likely to occupy an intermediate regime between these extremes, and systematic speciation studies with POMs remain an open opportunity.

Ionic strength (*I*) is the second lever. At pH 8, raising *I* in Tris–HCl (e.g., by NaNO_3_) suppressed the formation of highly charged fragments and shifted distributions toward intact Wells–Dawson cores, whereas the same POMs in sodium phosphate at higher *I* showed markedly less parent. Thus, TrisH^+^ ionic strength can be tuned to stabilize targeted species, while other buffers at comparable *I* bias decomposition or different equilibria. Finally, note that in some systems TrisH^+^ acts as a counterion only (no covalent engagement; cf. crystallographic salts), but even then, outer‐sphere pairing and H‐bond networks can influence solvation and assembly, subtly shifting speciation landscapes.

Implications and guidance. First, log the pH after mixing, not just the buffer setpoint: in Tris, the operational pH is the chemistry. Second, report ionic strength explicitly and consider salt complements when comparing across buffers. Third, when you see “more species” in Tris, do not equate it with more decomposition: it often indicates greater interaction and richer, controllable equilibria. Fourth, if the aim is to preserve a parent Wells–Dawson or Keggin in biotesting, moderate Tris with tuned ionic strength can outperform a “strong” buffer. Conversely, if you seek reactivity or derivatization, exploit Tris's interactive regime to access adduct‐stabilized or early intermediate states.

## 6 Conclusion

Tris is not a passive medium for polyoxometalates but a chemically active design element. Across the cases assembled here, Tris (i) installs μ‐O─CH_2_ linkages within POM scaffolds to create robust hybrids without prior core protonation, (ii) chelates and caps nascent tungsten‐oxo fragments to arrest early condensation and expose minimal, otherwise transient iso‐W motifs, and (iii) serves as a structure‐directing buffer that gates heterometal insertion and selects discrete architectures such as the Cr‐incorporated Keggin that displays single‐ion magnet behavior. The speciation atlas further shows that Tris–HCl does not simply “hold pH,” it shapes equilibria through pH drift, adduct formation, and ionic‐strength tuning. The same buffer‐as‐reagent logic extends beyond POMs: in metal–oxo clusters, MOFs/enzyme@MOF systems, and sol–gel‐derived oxides or nanoparticles, Tris and related amine buffers can coordinate transient nodes, template condensation, shift redox access, and modulate surface charge and colloidal stability—thereby steering both structure and function.

These findings motivate a speciation‐first mindset for any POM study in aqueous media, particularly in biology and formulation science. Report the operational pH after mixing, buffer identity and concentration, and ionic strength, confirm species with orthogonal analytics. For synthesis, treat Tris as a controllable variable rather than an unavoidable background: vary heterometal, face hydroxo pattern, and temperature profile to open or close alkoxylation windows, pair Tris with salts to modulate assembly and persistence.

For biologically oriented applications, Tris‐mediated speciation has direct implications for POM–membrane interactions, protein binding, and cytotoxicity. Changes in nuclearity, charge state, and surface functionalization in Tris buffers will alter electrostatic attraction to lipid interfaces, the ability of POMs to insert into or disrupt membranes, and their binding modes to proteins [42]. Formation of Tris adducts or TrisH^+^ ion pairs can partially shield charge patches, introduce additional hydrogen‐bond donors, or compete with protein ligands at metal or oxo sites. Moreover, Tris itself is known to interact with proteins [6] and to affect bacterial outer‐membrane permeability [5], so assays carried out in Tris always probe the combined response of the POM–Tris ensemble rather than the polyanion alone. We therefore recommend that antibacterial, antiviral, and anticancer studies include Tris‐only controls, compare at least one alternative buffer, and verify POM speciation under the exact assay conditions before assigning biological effects solely to a given POM structure.

Looking forward, four avenues appear most promising. First, Tris analogues such as triethanolamine (TEA), bis‐Tris propane, and tris(2‐hydroxypropyl)amine, which alter p*K*_a_, sterics, and triol geometry could deliver selective face capping, redox biasing, or orthogonal handles for bioconjugation. TEA has already been extensively exploited, particularly by Cronin and co‐workers [43], as a structure‐directing and ligating amine–triol for Mo/W cluster formation. Revisiting TEA in a speciation‐aware framework alongside Tris would allow us to decouple triol versus amine contributions and to map how a softer, less basic tertiary amine reshapes condensation, stability, and interfacial behavior. By contrast, bis‐Tris propane could strengthen multidentate capping but narrow the pH window. Second, structure‐directed heteroatom insertion under Tris control should be generalizable beyond chromium by targeting ions with comparable hydrolysis kinetics and accessible tetrahedral/octahedral switches—Al^III^, Ga^III^, Fe^III^, Co^III^, allowing rational access to other function‐bearing POMs. Third, mapping buffer‐specific speciation under realistic biomedia conditions (proteins, osmolytes, competing amines) will align laboratory synthesis with application in environments and prevent misattribution of biological effects. Fourth, translating these design rules to MOFs and sol–gel networks could yield predictable routes to biohybrid catalysts, stable dispersions, and targeted surface chemistries. In short, by recognizing buffers as reagents and Tris as architect, when it builds or selects structure, and gatekeeper, when it controls which species persist or interconvert by setting the operational pH, researchers gain both mechanistic clarity and synthetic leverage across the POM landscape and, more broadly, across buffer‐sensitive inorganic and hybrid materials.

## Conflicts of Interest

The authors declare no conflicts of interest.

## Data Availability

Data sharing is not applicable to this article as no new data were created or analyzed in this study.
